# Sensory Wheel and Lexicon for the Description of Cold-Pressed Hemp Seed Oil

**DOI:** 10.3390/foods12030661

**Published:** 2023-02-03

**Authors:** Matilde Tura, Mara Mandrioli, Enrico Valli, Caterina Dinnella, Tullia Gallina Toschi

**Affiliations:** 1 Department of Agricultural and Food Sciences, Alma Mater Studiorum—Università di Bologna, 40127 Bologna, Italy; 2 CIRI—Agrifood (Interdepartmental Centre of Industrial Agrifood Research), Alma Mater Studiorum—Università di Bologna, 47521 Cesena, Italy; 3 Department of Agriculture, Food, Environment and Forestry (DAGRI), University of Florence, 50144 Florence, Italy

**Keywords:** quality control, color, aroma and flavor, sensory evaluation sheet, descriptive analysis

## Abstract

Cold-pressed hemp seed oil (CP-HSO) has become available on the market and is gaining popularity mainly for its appeal and nutritional profile. The sensory quality largely depends on seed quality and processing as well as oil storage conditions. Given the “native” nature of the product, obtained by cold-pressing, the development of a standardized methodology to evaluate and describe the sensory quality of HSOs is of the utmost importance. To this aim, 16 commercial HSOs were evaluated, covering the main differences in brands and sales channels. A trained panel developed a vocabulary to describe the HSO profile consisting of 44 attributes, and a practical sensory wheel was proposed to classify attributes in different clusters and according to sensory modality. A sensory profile sheet was developed including two color descriptors (yellow, green), seven main positive (sunflower/pumpkin seeds, nutty, toasted nutty, hay, sweet, bitter, and pungent), several secondary positive (herbs, coffee, tobacco, etc.), four main defects (rancid, paint, burnt, and fish), and other secondary negative descriptors (boiled vegetables, cucumber, etc.). Subsequently, specific training of the panelists was carried out, and a satisfactory performance level was reached. This study represents the first attempt to standardize the sensory quality and terminology of HSO.

## 1. Introduction

Intense interest is increasing for cold-pressed (CP) oils, and their presence in the market is continuously growing [1]. Currently, the most common CP oils are from olive, sunflower, palm, rapeseed, and soybean, while hemp seed, flaxseed, and pumpkin are less frequent sources [2]. The global CP oil market is expected to increase from USD 24.62 billion (2018) to USD 36.40 billion (2026), with an annual growth of 5.3% [2]. According to The Codex Alimentarius, vegetables’ CP oils are defined as those “obtained, without altering the oil, by mechanical procedures only, e.g., expelling or pressing, without the application of heat. They may have been purified by washing with water, settling, filtering and centrifuging only” [3]. The cold-press process allows the preservation of minor compounds that are responsible for the richer sensory profile and higher antioxidant and pro-healthy activities of CP compared to refined oils [4]. According to Commission Regulation (EU) 2022/1393 [5], cold-pressed hemp seed oil (CP-HSO) is considered as food derived from hemp seeds, which are the seeds from the industrial type of *Cannabis sativa* L. The Reg. EU 2022/1393 established the maximum level of delta-9-tetrahydrocannabinol (Δ^9^-THC) equivalents for CP-HSO, i.e., 7.5 mg/kg. CP-HSO is a rich source of polyunsaturated fatty acids (PUFAs) [6] with a ω6:ω3 ratio of around 2.5–3:1, and it is considered optimal from a nutritional point of view and recommended for healthy diets [7,8]. CP-HSO also contains several minor bioactive compounds, such as tocopherols, which are powerful antioxidants and contribute to the prevention of cardiovascular disease [9].

A large variety of terpenes, mainly monoterpenes and sesquiterpenes, have been identified in different cannabis plant varieties [10] that can contribute to the aroma and flavor of CP-HSO together with other volatiles such as ketones, alcohols, esters, and aldehydes [4]. Aldehydes, ketones, esters, and furan derivatives resulting from biochemical transformations taking place during the oil production process and storage [4,11] can be responsible for off-flavors, such as rancid, which negatively impact odor notes, thus lowering both oil quality [12,13] and consumer acceptance [13]. Several sensory descriptors have been reported to describe the positive (nutty, aromatic, green, and herbaceous) and negative (fishy and painty) flavors, taste (bitter), and appearance (dark green, light green, greenish-yellow, olive-like, clear green, and yellow colors, transparency) of CP-HSOs [14,15,16,17,18,19]. Moreover, research on the sensory evaluation of cold-pressed hemp seed oil performed in a sensory laboratory and remotely has been recently published [20]. However, there is a paucity of information on the methodology used to identify sensory descriptors and a lack of consensus on the descriptor definition and valence for oil quality.

A large number of descriptive methods have been developed and applied since the 1950s. Among these, the quantitative descriptive analysis (QDA) remains the most mature and sophisticated sensory technique due to the possibility to perform both qualitative and quantitative evaluations of the sensory profile of food products [21].

This descriptive method uses a conventional descriptive analysis, which has been successfully utilized to provide a sensory profile of olive oil [22], marine oils [12], sunflower oil [23], liqueurs [24], and wines [25]. Moreover, several studies have proposed the use of a sensory wheel as a virtual, attractive, and useful tool to summarize the different sensory characteristics of a certain food, which can be effectively used in quality assessment, especially for training judges and keeping attributes in mind [12,26,27,28,29,30,31,32,33]. In fact, the sensory wheel is a practical visual tool that can be effectively used to describe the peculiar characteristics of samples. The combination of the QDA and sensory wheel can be useful to identify specific sensory attributes of the samples assessed and to visualize the sensory profile of each sample [31].

The aim of the present work was to establish an overall methodology for the evaluation of the sensory profile of CP-HSO by descriptive qualitative and quantitative approaches, as a crucial lever to define, recognize, and valorize high-quality CP-HSO.

In brief, a descriptive analysis was applied to 16 commercial CP-HSOs: (i) to identify the sensory descriptors; (ii) a sensory wheel was developed for attribute classification according to the sensory modalities; and (iii) a sensory evaluation sheet for the quality control was proposed.

## 2. Materials and Methods

### 2.1. Samples

Sixteen CP-HSO samples from the market, representative of the main brands and sales channels, were used. Fourteen samples were organic, while for two the farming system was not specified on the label, and all were packed in closed amber glass bottles (250 or 500 mL) and stored at room temperature, protected from direct light, until evaluation. All the samples were labeled as “cold-pressed”, and for two it was also specified that the extraction had been performed “without the use of solvents”. Independent replicates were performed by opening a different bottle of the same batch. In fact, due to the high sensitivity to oxidation of this oil, it was decided not to use the opened bottles to avoid differences in terms of rancidity.

### 2.2. Panel Selection

Nine assessors (5 men, age 27–56 years) were recruited from the staff, Ph.D. students, and research fellows at the Department of Agricultural and Food Science (Alma Mater Studiorum—Università di Bologna). All had previous experience and training in the sensory descriptive analysis of different food products and passed the test for physiological suitability for tasting virgin olive oils, according to the EU regulation 2568/91 and subsequent amendments [34].

### 2.3. Consensus on the Sensory Vocabulary

Assessors participated in 4 sessions to generate terms according to the descriptive analysis [35,36]. Assessors were asked to evaluate four samples of CP-HSO in each session and to freely describe their appearance, aroma, taste, flavor, and trigeminal and tactile sensations. Panelists were encouraged to use associative and cognitive terms rather than affective ones. At the end of each session, the panel leader listed all the elicited terms and took note of the occurrences of each term. The terms were grouped together on a semantic basis, and redundant terms were eliminated. Terms were classified into 12 classes and as positive or negative for oil quality according to the consensual decision of the panel. The consensus-building process, managed by the panel leader, ended with the list of attributes reported in Table 1.

### 2.4. Evaluation Sheet

**Figure 1:**
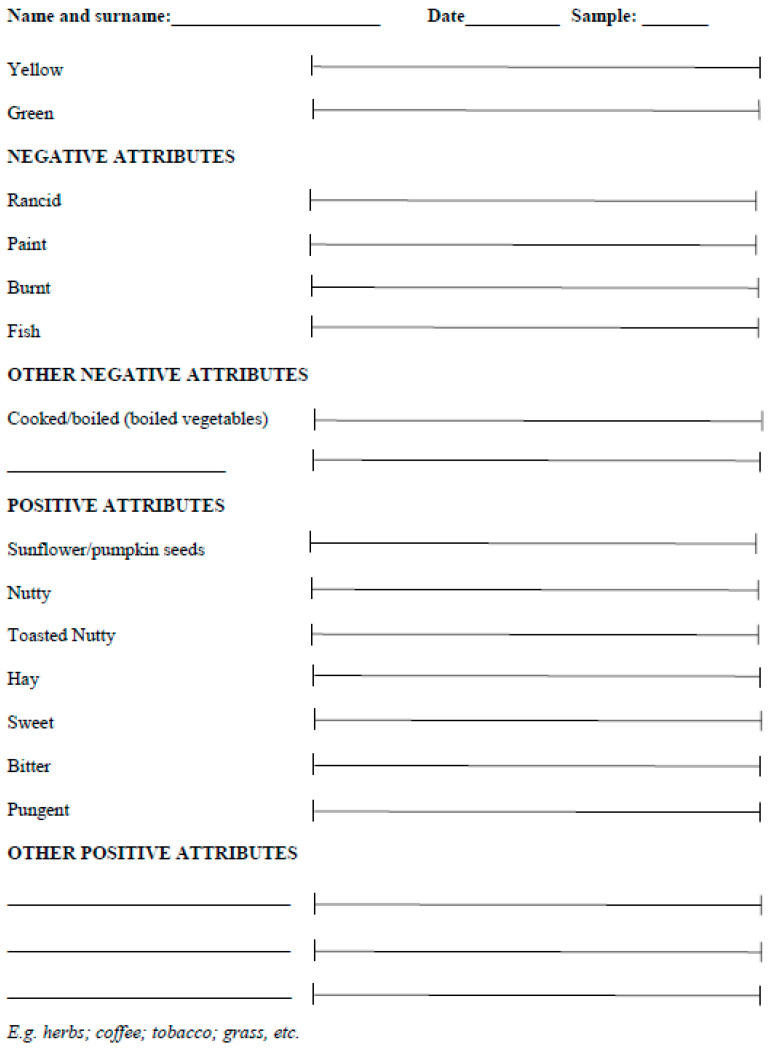
Sensory profile sheet specifically developed for cold-pressed hemp seed oils. The intensity of each attribute is evaluated with the use of a 100 mm unstructured scale with two anchor points: 0 (not perceivable) and 100 (extremely high).

Thirteen attributes were selected for inclusion in the sensory sheet according to the frequency of elicitation [28]. In particular, only the attributes for which there was a frequency of elicitation ≥5% were included in the sensory evaluation sheet. Thus, 13 descriptors were used for the construction of the profile sheet, and were classified, according to the panel, into the following: 4 main defect references (rancid, paint, burnt, and fishy) and 7 positive notes (sunflower/pumpkin seeds, nutty, toasted nutty, hay, sweet, bitter, and pungent). Several attributes, such as boiled vegetables, were grouped in the secondary negative descriptors, while grass, coffee, and tobacco references were identified as positive. Colors (yellow and green) were not classified as negative or positive. The evaluation sheet developed for the evaluation of the sensory profile of cold-pressed hemp oils is shown in Figure 1. The evaluation of the intensities and the training of the panel were performed on these 13 descriptors, while the others, i.e., the attributes for which a frequency of elicitation lower than 5% was highlighted, were included as “secondary positive attributes” or “secondary negative attributes” in the profile sheet. It was decided to construct a sensory profile sheet structured in a similar way to that used for the sensory evaluation of virgin olive oils (IOC/T.20/Doc. No 15) [39], since the tasters were already very familiar with this profile sheet, and all had experience in tasting virgin olive oils. The panel also suggested reporting secondary positive and negative attributes on the sensory profile sheet in order to describe the sample more thoroughly.

Reference standards were developed for each of the 13 descriptors to facilitate the consensus and calibrate the assessors. The standards, described in Table 1, were prepared to replicate moderate to high intensity. Reference CP-HSO samples were provided as a standard for yellow (three references corresponding to 20, 40, and 60 on the scale) and green (three references corresponding to 20, 60, and 80 on the scale).

### 2.5. Training Procedure

The panelists participated in 16 training sessions (corresponding to around 24 h) on rating intensities. During these sessions, the reference materials were presented to each assessor to develop a proper recognition of attributes in samples and the ability to rate their intensities on an unstructured scale. The assessors were asked to taste three different hemp seed oils, identify attributes, and rate their intensities on a 100 mm unstructured scale with two anchor points, namely 0 (extremely weak) on the left and 100 (extremely strong) on the right. It was decided to evaluate a maximum of 3 samples per session because some of the products showed very intense sensory characteristics: by increasing the number of samples, there could be the risk of sensory fatigue. The panelists were instructed to observe the color and smell flavor standards in order to help identify and rate the relevant sensations in the HSO samples. The entire evaluation procedure was performed according to ISO 13299:2010 [40]. Disposable white plastic glasses, coded with random three-digit codes, were used to taste the samples, pouring out around 15 g of oil. The presentation of the samples was randomized among assessors using a balanced Latin square design. Assessors were asked to take a sip and rate the descriptors’ intensity on the paper evaluation sheet.

Tasters were also advised to follow several rules before the evaluation, such as not smoking or drinking coffee at least 30 min before the test, in addition to the other indications given by the International Olive Council (IOC) for the assessment of virgin olive oil [39] and by the ISO 6658:2017 [41]. The samples were tested at room temperature. First, assessors were asked to analyze the samples visually. Subsequently, the tasting procedure was the same as that reported by the IOC for the assessment of virgin olive oil [39], with the only exception being that disposable glasses were used. For this reason, before the olfactory and gustatory phase, the panelists were asked to warm the sample by holding the glass in their hands, covering it, and rolling it.

Monitoring of the training of the panel was carried out using PanelCheck software (ver. 1.4.2; Nofima, Trømso, Norway). During the training, 16 sessions were necessary to reach acceptable levels of reproducibility and repeatability by the panelists. In particular, during the training sessions, the panel had difficulties related to the evaluation of color. For this reason, it was decided to present three different references for each color (yellow and green) (Table 1) and to assess the samples again until the misalignment was no longer present.

### 2.6. Data Analysis

Intensity data from the trained panel were elaborated by PanelCheck software (ver. 1.4.2; Nofima, Trømso, Norway), an open-source software that may be downloaded for free at http://www.panelcheck.com, accessed on 22 December 2022). In particular, a three-way ANOVA was performed to assess the importance of each descriptor in detecting the significant sensory differences among samples. Only significant attributes (*p* ≤ 0.05) were considered for further analyses. To graphically visualize the sensory profile of samples, the average scores for each discriminant attribute for each sample were determined and reported as spider plots using Excel (version 1808, Microsoft 365, Redmond, WA, USA). Finally, a principal component analysis (PCA) was conducted on the score of intensities of sensory descriptors using XLSTAT (version 2022.4.1) to understand the variance between samples and their attributes.

## 3. Results

### 3.1. Descriptors of Sensory Properties of CP-HSO

Assessors elicited a total of 55 descriptive terms for HSO sensory properties (Table 2). Consensus was reached on merging the following terms: drying oil with paint; smoked with burnt; toasted seeds with toasted; coffee ground with coffee; dark green and olive green with green; and walnut kernel with walnut; and eliminating esparto, fruity, heated, and salty. Thus, a final list of forty-four descriptors with associated definitions was obtained (Table 1) including three colors, two tastes, two mouthfeel sensations, and thirty-seven odor (aroma and flavor) attributes. The discussion led to the classification of 4 descriptors as main defects (rancid, paint, burnt, and fish) and 7 attributes as positive ones (sunflower/pumpkin seeds, nutty, toasted nutty, hay, sweet, bitter, and pungent). Several descriptors were indicated by the panel as secondary negative (boiled vegetables) or positive (herbs, coffee, tobacco, and grass) attributes. All 44 attributes, with the exception of the ones related to color, were classified by the panel as positive or negative, as reported in Table 2.

**Figure 2:**
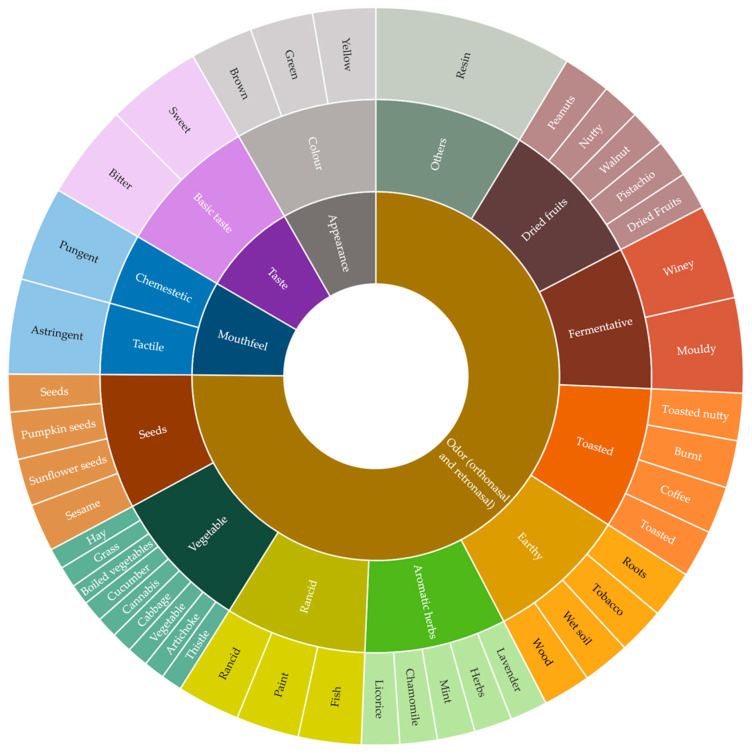
Sensory wheel for cold-pressed hemp seed oils.

Aroma and flavor attributes were then further classified in categories, with two describing the quality (rancid) and origin (fermented) of defects, and six describing the general aroma/flavor notes common to attribute sub-groups (earthy, toasted, seeds, dried fruits, aromatic herbs, vegetable). A sensory wheel organized in three levels was developed (Figure 2), where the outer level refers to the 44 attributes describing the sensory properties of CP-HSO; the middle level to aroma/flavor categories common to attribute sub-groups, taste, mouthfeel sensations; and the inner level to sensory modalities.

### 3.2. Profile Sheet for CP-HSO Tasting

Several descriptors reported by the panel were in line with what was previously published in the literature [14,15,16,17,18,19,42]. The most cited (>5%) attributes highlighted by the panel were as follows: yellow and green color, regarding appearance; seeds, nutty, toasted nutty, hay, rancid, fish, paint, burnt, sweet, bitter, and pungent in terms of olfactory and gustatory evaluation.

### 3.3. Evaluation of Panel Performances

**Figure 3:**
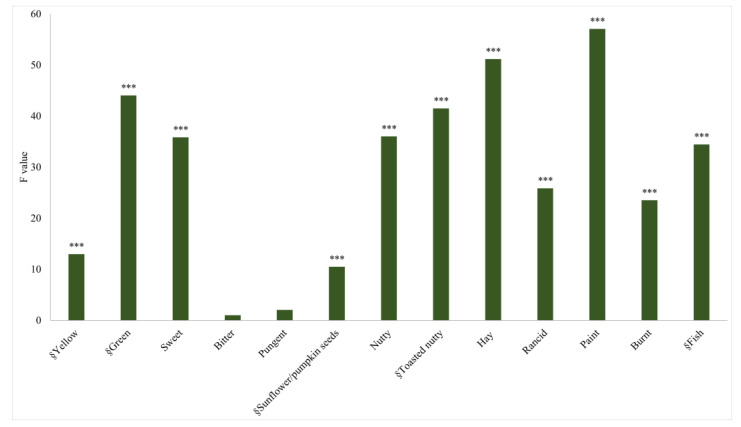
Three-way ANOVA on cold-pressed hemp seed oil (CP-HSO) intensity data: F-values of sample effect. Significance: *** indicates *p* < 0.001. § indicates that the F value was ×10^−1^.

Intensity data elaborated with PanelCheck software gave important information regarding the panel’s discriminatory ability, alignment, and reproducibility, following the workflow proposed by Tomic et al. (2010) [43]. The cold-pressed HSO evaluation showed a significant sample effect on 11 (yellow, green, sweet, sunflower/pumpkin seeds, nutty, toasted nutty, hay, rancid, paint, burnt, and fish) of 13 attributes (*p* ≤ 0.000), and only bitter taste (F value = 1.02, *p* = 0.057) and pungency (F value = 2.03, *p* = 0.057) did not discriminate among samples (Figure 3). No replicate effect was found (*p* ≥ 0.22), and the significant assessor effects showed F values that were much lower compared to the sample effect for all attributes and could be considered negligible.

**Figure 4:**
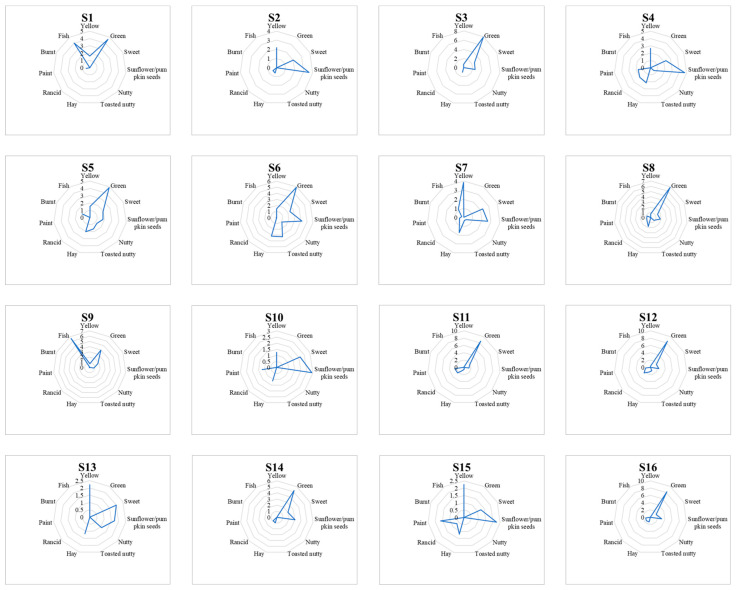
Spider graphs representing the sensory profiles of the 16 CP-HSOs (named from S1 to S16) analyzed.

Figure 4 shows the sensory profiles of the 16 CP-HSOs tested, which were very different, especially for color attributes; sample S7 showed the highest mean score for yellow, and S12 for green. In addition, samples differed in terms of positive and negative odor notes: sample S6 showed the highest mean score for the intensities of sunflower/pumpkin seeds, toasted nutty, and hay, and sample S11 showed the highest intensity mean scores for rancid and paint.

**Figure 5:**
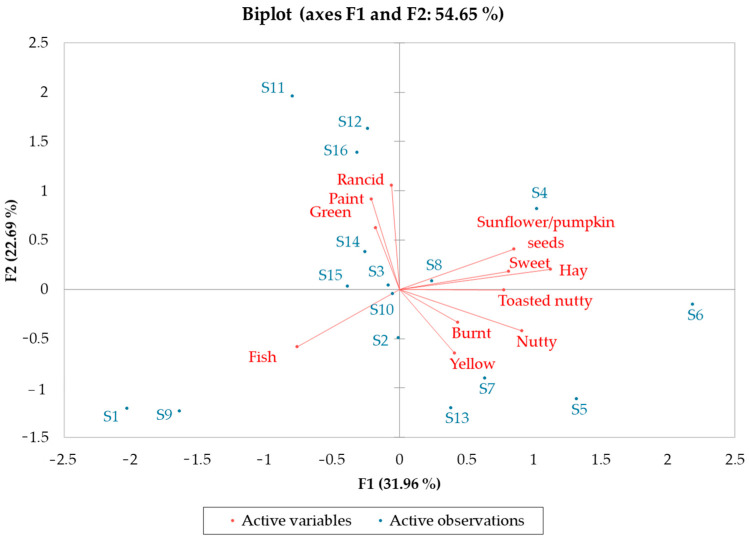
Principal component analysis plot of the intensities of sensory descriptors and samples of CP-HSO.

To gain additional information, a PCA was performed on the score of the intensities of the 11 discriminant descriptors for all 16 CP-HSOs tested (Figure 5). The first two principal components (PC1 and PC2) explained 54.65% of total variance. PC1 (31.96% of variance) was more related to positive and negative aroma and the taste component, since its axis was mainly formed by sweet, sunflower/pumpkin seeds, hay, toasted nutty, nutty, and burnt, and PC2 (22.69% of variance) to the color and rancidity components, since its axis was formed by green, yellow, paint, and rancid. Figure 5 highlights that samples S1 and S9 were mainly described by a fish note, and samples S11, S12, and S16 by paint and rancid notes, both of which can be related to the oxidative degradation of the samples [42,44].

## 4. Discussion

This study aimed to identify the sensory attributes of HSOs and organize them in a sensory wheel to be used as a training tool in quality assessment; moreover, an evaluation sheet for the HSO sensory profile was also developed. The selection of samples covering, as much as possible, the sensory variation of the target product represents a key aspect for the effective development of sensory descriptive vocabulary [12,45]. In the present study, 16 samples representative of the HSO, collected in the Italian market (EU or non-EU origin, available in the supermarkets, sector shops, and online) were evaluated. The number of samples is comparable to that used in other studies aimed at developing a sensory descriptive vocabulary for hibiscus tea (n = 22, [46]) and commercial chocolate samples (n = 5 white chocolates, n = 8 milk chocolates, and n = 9 dark chocolates, [28]). The sensory vocabulary included 44 attributes that were specific to CP-HSO, and also attributes with a low citation frequency were retained in order to cover the possible sensory space, thus allowing for a detailed description of CP-HSO, including defects. With the same inclusive approach, descriptors were depicted in a sensory wheel (Figure 2), without applying a rigid reduction of the number of attributes, in order to avoid the possible loss of terms that could be essential in defining the unique sensory profiles of the product [12]. Thus, the first sensory wheel for CP-HSO proposed herein can be used as a disseminating tool between researchers, producers, technicians, and other key players to share and disseminate the sensory lexicon, as a common reference, including each possible sensory peculiar note. It can also be a starting point to communicate and correctly advertise the sensory quality of CP-HSO to consumers. Additionally, the wheel and profile sheet can be applied to assess and compare the quality of different products, and to evaluate changes during the storage of oils and indicate food pairings with specific food products, food dishes, and CP-HSOs. Future research will be necessary to possibly develop variety-specific sensory wheels, which can be useful to distinguish monovarietal CP-HSOs, if needed. Some of the attributes identified by the panel and reported in the sensory wheel have already been identified in the previous literature, such as the smell of paint in strongly oxidized hemp seed oils, as well as a hint of fish [42], rancid, and fish sensory notes [12], also attributable to an advanced oxidative process. The category of rancid defect was mainly described by the terms fishy and paint. The fishy notes may be due to the presence of sulfur-containing compounds, such as dimethyl trisulfide or trimethylamine, or to the fungal infection of seeds that are not correctly dried [42,47]. The paint negative attribute could be related to the oxidation of free fatty acids [42], which can lead to the formation of volatile secondary oxidation products, such as aldehydes and aliphatic ketones [48]. In particular, according to the literature, the paint attribute is caused by saturated and unsaturated aldehydes, including hexanal, (*E,Z*)- and (*E,E*)-2,4-heptadienals and (*E,E*)- and (*E,Z*)-2,4-decadienal [49]. In particular, it was found that (*E,Z*)-2,4-heptadienal is one of the volatile oxidation products for which formation in greater quantities in hemp oils subjected to forced oxidation (60 °C) for 18 days has been demonstrated. Therefore, it is undoubtedly a compound that strongly characterizes the sensory rancidity of hemp oil [50]. Moreover, the paint sensory descriptor has been previously related to an increase in peroxide and anisidine values in a study carried out on fish oil and microencapsulated fish oils [51]. It is well known that the oxidation of linoleic acid leads to the formation of hexanal, 2-heptenal, 2-octenal, (*E,Z*)-2,4-decadienal, and (*E,E*)-2,4-decadienal, while starting from linolenic acid, (*E,Z*)-2,4-heptadienal and (*E,E*)-2,4-heptadienal are usually produced [48]. Several of these compounds were previously related to off-flavors, such as (*E,E*)-2,4-decadienal with deep-fried notes, (*E*)-2-heptenal with oxidized aroma, and 2,4-heptadienal with rancid [44]. According to the literature, freshly produced cold-pressed hemp seed oil is characterized by notes of citrus, mint, and pepper [42]. Citrus and mint notes can be given by limonene, while pepper is related to β-caryophyllene [52]. In addition, the assessors identified mint notes among these descriptors. It is important to underline that sensory characteristics can vary according to the variety of seeds; environmental conditions of their growth; and how the seeds are dried, stored [42], and processed [53]. The flavor of CP-HSO is linked to the presence of terpenes in the aromatic profile [54]. For this reason, a crucial point of the production process is drying hemp seeds: it is essential to dry them slowly at low temperatures (<25 °C) to a target moisture content <10% in order to preserve the terpenic fraction and prevent the formation of off-flavors related to oxidation, such as those reminiscent of jute sacks or jute rope, but also paint or fish [42].

Starting from the frequency of descriptors cited, a sensory profile sheet was also developed. In particular, the selected terms were as follows: yellow, green, rancid, paint, burnt, fish, sunflower/pumpkin seeds, nutty, toasted nutty, hay, sweet, bitter, and pungent. Moreover, according to the panel, free space in the profile sheet was left after the main positive and negative attributes to indicate other descriptors, such as those reported in the sensory wheel. The free space was left for two reasons: (*i*) to highlight the unique sensory characteristics of the CP-HSO linked, e.g., with processing or hemp variety; and (*ii*) to gain an attitude of the panelists that, being all tasters of virgin olive oils, was used to seek additional secondary positive attributes. The descriptors written in the sensory profile sheet allowed discrimination among samples, as reported in Section 3.3, except for bitter and pungent for which the *p*-value was slightly higher than 0.5, thus indicating the goodness of the vocabulary generated. Eventually, the sensory profile sheet presented herein could be used to assess the sensory quality of CP-HSOs, e.g., for the industry to perform internal quality control and define and predict its sensory shelf-life.

## 5. Conclusions

To date, and to the authors’ knowledge, the present investigation is the first dealing with the sensory evaluation of CP-HSOs and the first presenting a sensory wheel. In particular, the lexicon developed for hemp seed oils includes 44 descriptors, consisting of 3 visual attributes, 2 taste attributes, 1 tactile sensation, 1 trigeminal sensation, and 37 odor attributes. The wheel is proposed as an effective and practical tool to train assessors in terms of lexicon and descriptors. Moreover, a specific qualitative and quantitative descriptive sensory evaluation sheet for CP-HSO was developed to train a panel and to be adopted for the evaluation of the sensory quality of commercial CP-HSOs. In the future, it will be important to define a relation between olfactory sensory descriptors and volatile compounds, with the aim of producing reproducible sensory and instrumental reference standards.

## Figures and Tables

**Table 1:** Consensus list of attributes describing appearance, aroma, flavor, taste, and mouthfeel sensations of samples. Attributes in bold were used to construct the sensory profile sheet for cold-pressed hemp seed oils.

Attribute	Definition	Standard	Anchor Point (Intensity on a 100 mm Unstructured Scale)
*Appearance*			
Brown	Intensity of brown color	-	
**Green**	Intensity of green color	A selected cold-pressed hemp seed oil 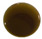 A selected cold-pressed hemp seed oil 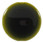 A selected cold-pressed hemp seed oil 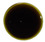	20/100 60/100 80/100
**Yellow**	Intensity of yellow color	A selected cold-pressed hemp seed oil 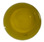 A selected cold-pressed hemp seed oil 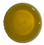 A selected cold-pressed hemp seed oil 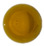	20/100 40/100 80/100
*Taste*			
**Bitter**	Taste associated with caffeine, chicory, tonic water [37]	Caffeine 2.0 g/L in water	50/100
**Sweet**	Taste associated with sucrose [37]	Sucrose 12.0 g/L in water	100/100
*Mouthfeel*			
Astringent	Dryness of the oral surface and tightening and puckering sensation of the mucosa and muscles around the mouth [37]	-	
**Pungent**	Sensation of tingling perceived in the oral cavity [38]	Capsaicin 0.8 mg/kg in water	100/100
*Aroma and flavor*		-	
*Aromatic herbs*			
Camomile	Olfactory sensation of chamomile flower	-	
Herbs	Olfactory sensation of aromatic herbs reminiscent of balsamic	-	
Lavender	Olfactory sensation of lavender flower	-	
Liquorice	Olfactory sensation of liquorice roots	-	
Mint	Olfactory sensation of mint leaf	-	
*Dried fruits*			
Dried fruit	Olfactory sensation reminiscent of a mix of dried fruits (i.e., nuts, walnuts, peanuts)	-	
**Nutty**	Olfactory sensation of fresh hazelnuts	10 g of fresh hazelnut in a disposable glass	100/100
Peanuts	Olfactory sensation of dried peanuts	-	
Pistachio	Olfactory sensation of dried pistachios	-	
Walnut	Olfactory sensation of shelled walnuts	-	
*Earthy*			
Roots	Olfactory sensation of earth, soil	-	
Tobacco	Olfactory sensation characteristic of dried tobacco	-	
Wet soil	Olfactory sensation of oil obtained from seeds that have been collected with earth or mud on them and which have not been cleaned	-	
Wood	Olfactory sensation of damp wood	-	
*Fermentative*			
Moldy	Olfactory sensation of oils obtained from seeds in which large numbers of fungi and yeasts have developed as a result of its being stored in humid conditions for several days	-	
Winey	Olfactory sensation of certain oils reminiscent of wine	-	
*Rancid*			
**Fish**	Olfactory sensation of fish oil	0.5 g/20 mL of fish oil in a disposable glass	50/100
**Paint**	Olfactory sensation of paint, siccative oils, linoleum	A selected cold-pressed hemp seed oil subjected to a forced oxidation (Rancimat/Oxidative Stability Instrument, 24 h at 110 °C) in a disposable glass	100/100
**Rancid**	Olfactory sensation characteristic of strongly oxidized oils or fats	International Olive Oil Council standard for rancid of olive oil	90/100
*Seeds*			
Pumpkin seeds	Olfactory sensation of pumpkin seeds	-	
**Seeds**	Olfactory sensation of seed mixes (pumpkin and sunflower)	Mixture of 50% of pumpkin seeds and 50% of sunflower seeds in a disposable glass	100/100
Sesame	Olfactory sensation of sesame seeds	-	
Sunflower seeds	Olfactory sensation of sunflower seeds	-	
*Toasted*			
**Burnt**	Olfactory sensation of burnt seeds	Mixture of burnt sunflower seeds, hazelnuts, and pumpkin seeds (cooked in the oven at 200 °C for 1 h) in a disposable glass	100/100
Coffee	Olfactory sensation characteristic of coffee		
Toasted	Olfactory sensation of toasted note reminiscent of toasted cereals	-	
**Toasted nutty**	Olfactory sensation of toasted hazelnut	Toasted hazelnuts (cooked in the oven at 150 °C for 25 min) in a disposable glass	100/100
*Vegetables*			
Artichoke	Olfactory sensation characteristic of artichoke	-	
Boiled vegetables	Olfactory sensation of boiled vegetable (such as chicory and savoy cabbage)	-	
Cabbage	Olfactory sensation characteristic of boiled cabbage	-	
Cannabis	Olfactory sensation characteristic of cannabis plant	-	
Cucumber	Flavor produced when an oil is hermetically packed for too long, particularly in tin containers, which is attributed to the formation of 2,6-nonadienal	-	
Grass	Olfactory sensation characteristic of freshly mown grass	-	
**Hay**	Olfactory sensation of hay	Dried hay in a disposable glass	100/100
Thistle	Olfactory sensation characteristic of thistle	-	
Vegetable	Olfactory sensation characteristic of fresh broad-leaved vegetables	-	
*Other*			
Resin	Olfactory sensation of natural resin or pine resin	-	

**Table 2:** Sensory attributes in alphabetic order generated through the language development sessions.

Attribute	Frequency of Elicitation (%)	Class
Brown (V)	0.7	Color
Dark green (V)	0.1	
**Green (V)**	7.2	
Olive green (V)	0.4	
**Yellow (V)**	8.7	
**Bitter** ^1^ **(T)**	5.0	Basic taste
Salty (T)	0.1	
**Sweet** ^1^ **(T)**	6.7	
Astringent ^1^ (M)	0.4	Mouthfeel
**Pungent** ^1^ **(M)**	5.3	
Chamomile ^1^ (O)	0.4	Aromatic herbs
Herbs ^1^ (O)	0.6	
Lavender ^1^ (O)	0.3	
Liquorice ^1^ (O)	0.3	
Mint ^1^ (O)	0.1	
Dried fruit ^1^ (O)	0.2	Dried fruits
**Nutty** ^1^ **(O)**	5.0	
Peanuts ^1^ (O)	0.2	
Pistachio ^1^ (O)	0.2	
Walnut ^1^ (O)	0.1	
Walnut kernel (O)	0.1	
Roots ^2^ (O)	0.1	Earthy
Tobacco ^1^ (O)	0.1	
Wet soil ^2^ (O)	0.1	
Wood ^2^ (O)	0.6	
Esparto (O)	0.1	Fermentative
Moldy ^2^ (O)	1.3	
Winey ^2^ (O)	0.2	
Resin ^1^ (O)	0.1	Others
Drying oil (O)	0.3	Rancid
**Fish** ^2^ **(O)**	5.2	
Heated (O)	0.4	
**Paint** ^2^ **(O)**	5.1	
**Rancid** ^2^ **(O)**	11.0	
Pumpkin seeds ^1^ (O)	1.0	Seeds
**Seeds** ^1^ **(O)**	5.2	
Sesame ^1^ (O)	0.3	
Sunflower seeds ^1^ (O)	4.8	
**Burnt** ^2^ **(O)**	5.0	Toasted
Coffee ^1^ (O)	0.5	
Coffee grounds (O)	0.1	
Toasted ^1^ (O)	1.1	
**Toasted nutty** ^1^ **(O)**	5.3	
Toasted seeds (O)	0.1	
Smoked (O)	0.1	
Artichoke ^1^ (O)	0.1	Vegetables
Boiled vegetables ^2^ (O)	0.9	
Cabbage ^2^ (O)	0.2	
Cannabis ^1^ (O)	0.4	
Cucumber ^2^ (O)	0.5	
Fruity (O)	0.2	
Grass ^1^ (O)	1.6	
**Hay** ^1^ **(O)**	5.2	
Thistle ^1^ (O)	0.8	
Vegetable ^1^ (O)	0.5	

Underlined words represent the terms for which a consensus was not gained, and which were not used for construction of the sensory wheel. Bold text represents the odor (O) both by orthonasal and retronasal sensations, taste (T), visual (V), and mouthfeel sensation (M) attributes chosen for the sensory profile sheet. The percentage represents the frequency of elicitation of each attribute. The attributes were classified in 12 classes and as positive (^1^) or negative (^2^), with the exception of color attributes.

## Data Availability

The data are available from the corresponding author.
